# Prognostic value of lymphocyte-to-monocyte ratio among Asian lung cancer patients: a systematic review and meta-analysis

**DOI:** 10.18632/oncotarget.20574

**Published:** 2017-08-28

**Authors:** Wen Li, Guangzhi Ma, Qiang Wu, Yunfu Deng, Ya Liu, Jing Wang

**Affiliations:** ^1^ Department of Thyroid and Breast Surgery, West China Hospital, Sichuan University, Chengdu 610041, P.R. China; ^2^ Cancer Center, West China Hospital, Sichuan University, Chengdu 610041, P.R. China; ^3^ Department of Thoracic Surgery, West China Hospital, Sichuan University, Chengdu 610041, P.R. China

**Keywords:** lymphocyte to monocyte ratio (LMR), lung cancer, prognosis, meta-analysis

## Abstract

**Purpose:**

Numerous studies have reported the prognostic significance of lymphocyte-to-monocyte ratio (LMR) in malignancies, but its prognostic value among lung cancer remains controversial. This meta-analysis aimed to explore the prognostic significance of LMR in lung cancer patients.

**Results:**

Eight studies including 3954 patients were included in this meta-analysis. Pooled results indicated that low LMR was significantly associated with poorer progression-free survival (hazard ratio (HR): 1.431, 95% confidence interval (CI): 1.294–1.582, *p* < 0.001) and overall survival (OS) (HR: 1.651, 95% CI: 1.306–2.086, *p* < 0.001), compared with high LMR. Similar results were observed in subgroups regardless of treatment, LMR cut-off value, or districts. However, no significant correlation between the LMR and OS was observed in the small cell lung cancer (SCLC) subgroup (HR = 1.262, 95% CI: 0.864–1.841, *p* = 0.229).

**Materials and Methods:**

Identified literatures were extracted and retrieved from PubMed, Embase, Web of Science, and the Cochrane Library databases; All eligible studies focused on the association between LMR and the prognosis of lung cancer.

**Conclusions:**

Low LMR is associated with poor outcomes among lung cancer patients. Further studies are needed to discuss the correlation between LMR and lung cancer prognosis.

## INTRODUCTION

Lung cancer is one of the most commonly diagnosed cancer and the main cause of cancer morbidity worldwide [1]. Approximately 1.8 million new cases are diagnosed and causes around 1.4 million cancer-related deaths annually [2]. Although diagnosis and treatment strategy have improved in the past decades, the five-year survival rate of lung cancer remains unsatisfactory due to the risk of local recurrence or distal metastasis [3]. Studies have identified multiple prognostic factors in lung cancer patients [4], but promising markers that demonstrate prognostic value remain lacking [5]. Hence, identifying a novel potential biomarker useful in selecting appropriate treatment strategies and predicting prognosis is vital [6].

Systemic inflammatory response is correlated with formation and recurrence of various cancers [7–9]. Moreover, inflammation may cause the tumor microenvironments to promote cancer progression [10, 11]. Studies have recently demonstrated that lymphocyte-to-monocyte ratio (LMR) is a prognostic indicator in some cancers, including hepatocellular cancer, endometrial cancer, breast cancer, and gastrointestinal cancer [6, 12–15]. Although the prognostic significance of LMR in lung cancer patients have been evaluated, contradicting conclusions were drawn. Hu et al. found that high LMR is a favorable factor and associated with longer OS compared with low LMR [16]. However, Cao et al. found no association between LMR and OS in lung cancer [17]. Thus, we performed a meta-analysis to determine the prognostic value of LMR in lung cancer.

## RESULTS

### Literature search

**Figure 1:**
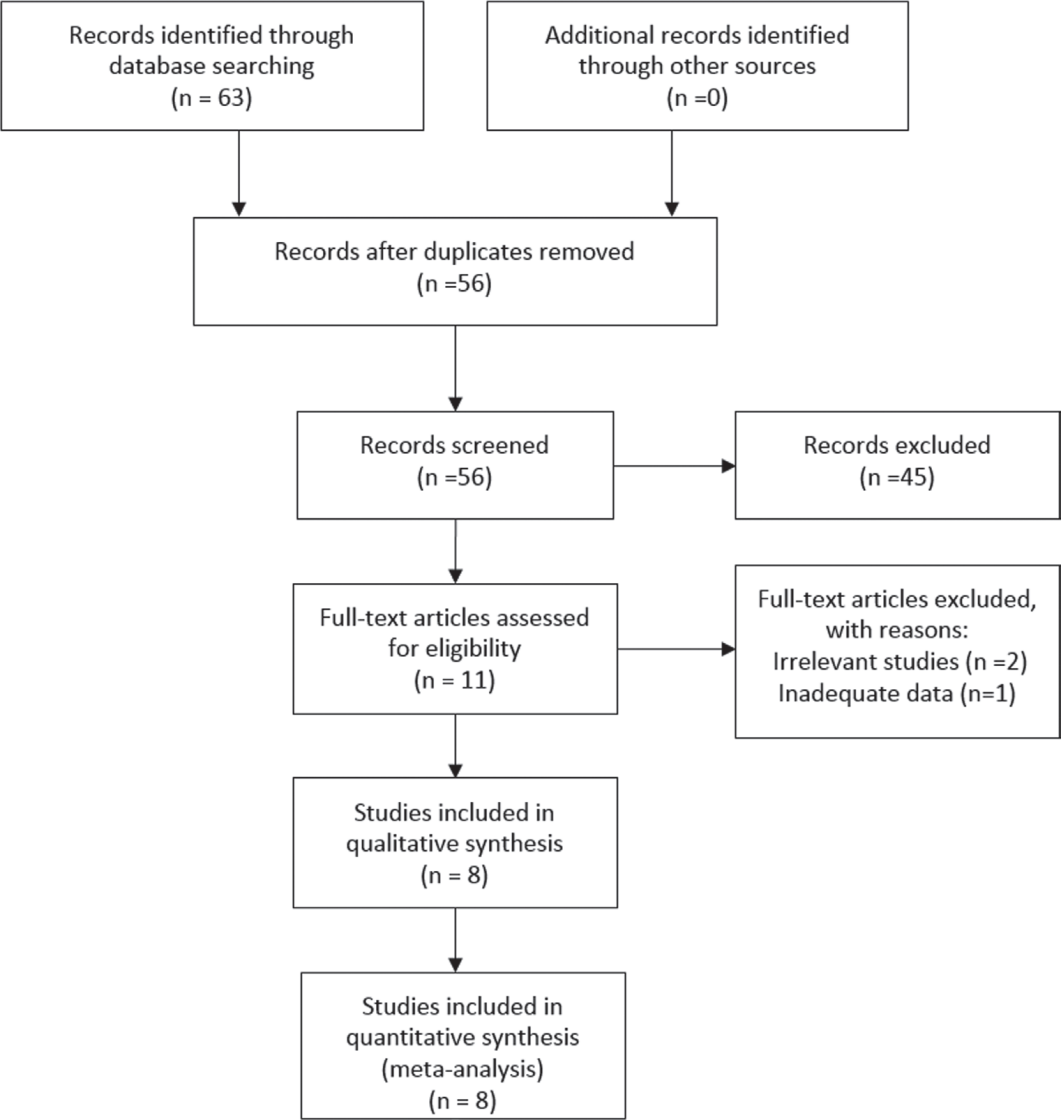
The selection process for eligible studies

Our search strategy involved screening of 63 potentially relevant papers (Figure 1). Based on their titles and abstracts, 11 studies were eventually assessed. After reading their full texts, eight studies with a total of 3954 patients were included in this meta-analysis [16–23].

### Characteristics of the eligible studies

The eight studies were retrospective studies, published between 2014 and 2017, and with a sample size of 74–1453 (Table 1). The patients’ median age ranged from 50 years to 69 years. As for ethnicity, all of the studies included Asian populations. The LMR cut-off values ranged from 2.62 to 4.56. Seven studies reported disease-free survival (DFS) or PFS, and all studies determined the OS. HRs and 95% CIs were determined directly from these studies. The NOS scores of the eight studies varied from 6 to 8.

**Table 1:** Characteristics of the included studies

Author	Year	Country	N of patients (F/M)	Median age	Histological subtype	Tumor stage	Treatment	Survival Reported	Cut-off value of LMR	HR estimation	NOS scores
Hu et al.	2014	China	1453 (418/1035)	59	NSCLC/SCLC	I–III	Surgery	DFS and OS	3.68	MA.	8
Lin et al.	2014	China	370 (157/213)	63.6	NSCLC	IIIB–IV	Non-surgery	DFS and OS	4.56	MA.	7
Go et al.	2014	Korea	188 (74/114)	69	SCLC	LD/ED/LHD	Non-surgery	PFS and OS	4.19	MA.	7
Chen et al.	2015	China	235 (131/104)	65.2	NSCLC	IIIB–IV	Non-surgery	PFS and OS	3.29	MA.	6
Wang et al.	2015	China	74 (39/35)	50	NSCLC	I–IV	Various	PFS and OS	3.82	MA.	6
Xia et al.	2016	China	439 (152/287)	62	NSCLC	I	Surgery	RFS and OS	4.00	MA.	8
Song et al.	2016	China	488 (129/359)	64	NSCLC	I–II	Surgery	PFS and OS	4.50	MA.	7
Cao et al.	2017	China	707 (253/454)	56.2	SCLC	LD ED	Non-surgery	OS	2.62	MA.	8

N: number; NSCLC: non-small cell lung cancer; SCLC: small cell lung cancer; LD: limited-stage disease; ED: extensive-stage disease; LDH: lactate dehydrogenase; DFS: disease-free survival; RFS: recurrence-free survival; OS: overall survival; MA: multivariate analysis; NOS: Newcastle-Ottawa Scale.

### Correlation between LMR and PFS

**Figure 2:**
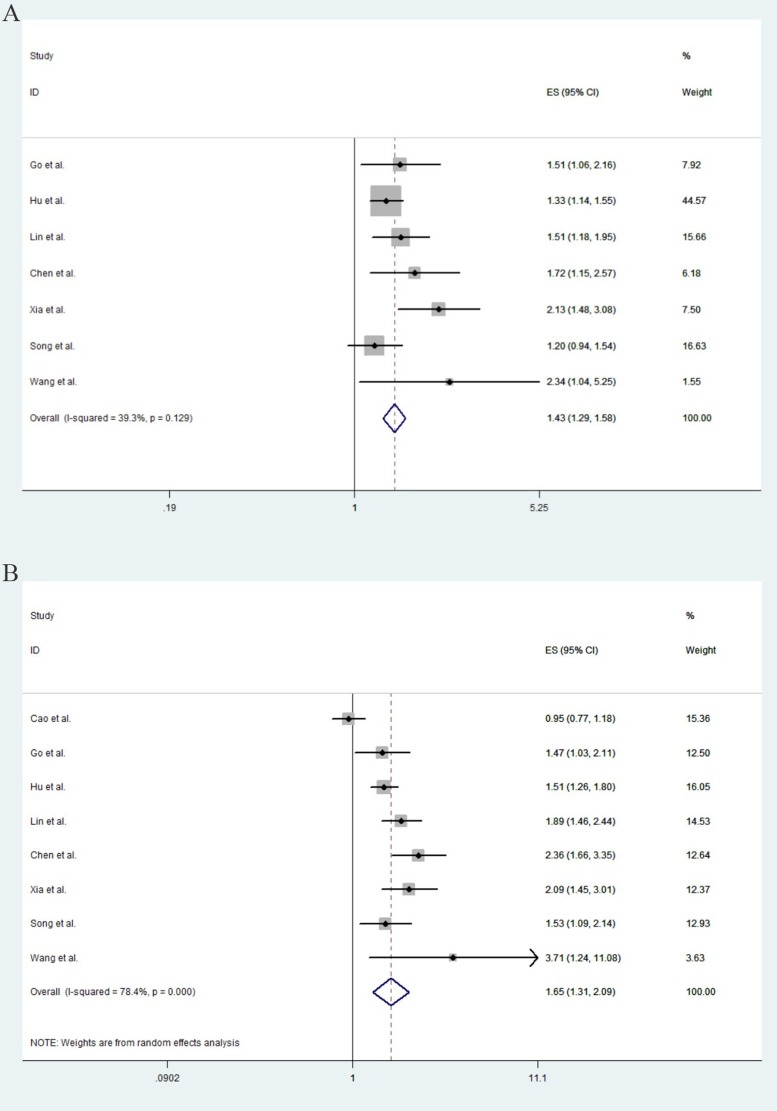
The pooled estimated survival (ES) (hazard ratios) for PFS (**A**) and OS (**B**) in Asian lung cancer patients with low LMR.

The effect of LMR on PFS was evaluated in seven studies, which include 3247 patients. The results showed that patients with low LMR displayed worse PFS than those with high LMR (HR = 1.431, 95% CI: 1.294–1.582, *P* < 0.001; Figure 2A). No significant heterogeneity (*I*^2^ = 39.30%, *p* = 0.129) was found and thus fixed-effect model was used.

### Correlation between LMR and OS

All of the eight studies were included in the analysis of OS. As shown in Figure 2B, lung cancer patients with low LMR displayed a significantly poorer OS than those with high LMR (HR = 1.651, 95% CI: 1.306–2.086, *p* < 0.001). A significant heterogeneity among these studies was observed (*I*^2^ = 78.40%, *p* < 0.001), and random-effect model was used.

### Subgroup analyses

The included studies were divided into subgroups according to the data extracted, as follows: (1) Pathology (NSCLC/SCLC); (2) Therapy (Surgery/Non-surgery); (3) LMR value (≥ 4/< 4); and (4) Chinese patients.

### NSCLC/SCLC

Six studies on NSCLC were conducted, and the pooled HRs for PFS and OS were 1.486 (CI: 1.269–1.740, *p* < 0.001, *I*^2^ = 54.30%) and 1.751 (CI: 1.553–1.975, *p* < 0.001, *I*^2^ = 43.60%), respectively. As for SCLC patients, OS was reported in three literatures, and the pooled result for OS was 1.262 (CI: 0.864–1.841, *p* = 0.229). Heterogeneity was significant (*p* = 0.04, *I*^2^ = 68.9%) hence random-effect model was applied.

### Surgery/non-surgery

Three studies focused on patients that underwent surgery. The combined HR for PFS was 1.443 (CI: 1.113–1.872, *p* = 0.006, *I*^2^ = 70.6%). The pooled OS was 1.593 (CI: 1.379–1.840, *p* < 0.001); no significant heterogeneity (*p* = 0.279, *I*^2^ = 21.6%) was found and fixed-effect model was used. With regard to non-surgery patients, the pooled PFS and OS were 1.553 (CI: 1.292–1.868, *p* < 0.001, *I*^2^ = 0.0%) and 1.563 (1.025–2.386, *p* = 0.038, *I*^2^ = 88.4%), respectively.

### LMR value

When LMR was no less than 4, the pooled HR for PFS and OS was 1.516 (CI: 1.213–1.893, *p* < 0.001, *I*^2^ = 32.9%) and 1.747 (CI: 1.488–2.05, *p* < 0.001, *I*^2^ = 0%). As for studies that reported LMRs less than 4, the combined HR for PFS was 1.394 (1.213–1. 602, *p* < 0.001, *I*^2^ = 78.1%) and the pooled HR for OS was 1.630 (1.053–2.521, *p* = 0.028, *I*^2^ = 87.80%).

### Chinese patients

Seven studies were conducted in China. The PFS reported in six studies were pooled, and the combined HR was 1.425 (CI: 1.283–1.582, *p* < 0.001, *I*^2^ = 49.00%). All literatures examined OS, and the pooled result was 1.687 (CI: 1.293–2.199, *p* < 0.001, *I*^2^ = 81.40%). All pooled results are shown in Table 2.

**Table 2:** Meta-analyses of correlation between LMR and survival of lung cancer patients

	*N* of studies	HR (95% CI)	Log-rank *P*	Heterogeneity (*p*, *I^2^*)
Total PFS	7	1.431 (1.294–1.582)	< 0.001	0.129,39.3%
NSCLC PFS	6	1.486 (1.269–1.740)	< 0.001	0.052,54.3%
SCLC PFS	1	1.509 (1.056–2.157)	0.024	—
Surgery PFS	3	1.443 (1.113–1.872)	0.006	0.033,70.6%
Non-surgery PFS	3	1.553 (1.292–1.868)	< 0.001	0.863,0.0%
Cut-off value ≥ 4 PFS	4	1.516 (1.213–1.893)	< 0.001	0.225,32.9%
Cut-off value < 4 PFS	3	1.394 (1.213–1.602)	< 0.001	0.01,78.1%
Chinese PFS	6	1.425 (1.283–1.582)	< 0.001	0.081,49.0%
Korean PFS	1	1.509 (1.056–2.157)	0.024	—
Total OS	8	1.651 (1.306–2.086)	< 0.001	< 0.001,78.4%
NSCLC OS	6	1.751 (1.553–1.975)	< 0.001	0.115,43.6%
SCLC OS	3	1.262 (0.864–1.841)	0.229	0.04,68.9%
Surgery OS	3	1.593 (1.379–1.840)	< 0.001	0.279,21.6%
Non-Surgery OS	4	1.563 (1.025–2.386)	0.038	< 0.001,88.4%
Cut-off value ≥ 4 OS	4	1.747 (1.488–2.050)	< 0.001	0.432,0%
Cut-off value < 4 OS	4	1.630 (1.053–2.521)	0.028	< 0.001,87.8%
Chinese OS	7	1.687 (1.293–2.199)	< 0.001	< 0.001,81.4%
Korean OS	1	1.472 (1.029–2.106)	0.034	—

*N*: number; HR: hazard ratio; CI: confidence interval; PFS: progression-free survival; OS: overall survival; NSCLC: non-small cell lung cancer; SCLC: small cell lung cancer.

### Publication bias

**Figure 3:**
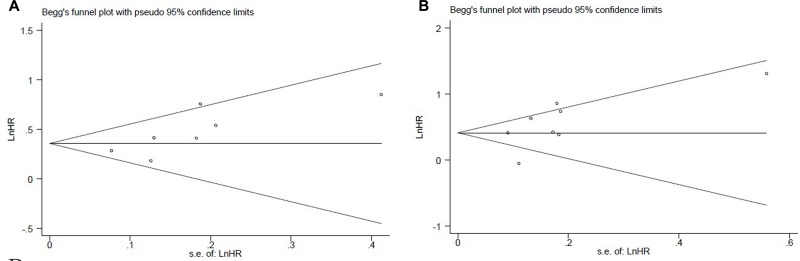
The Begg's publication bias plots of the studies that reported the correlation between low LMR and PFS (**A**) and OS (**B**) in patients that developed lung cancer.

Publication bias was not significant in the current meta-analysis based on the plots of publication shown in Figure 3.

## DISCUSSION

Inflammation is critical in tumor growth, invasion, and metastasis; many inflammatory indicators, including neutrophilocyte-to-lymphocyte ratio, derived neutrophilcyte-to-lymphocyte ratio, platelet-to-lymphocyte ratio, and LMR, are prognostic factors in various cancers [15, 19, 24–26]. This meta-analysis is the first to determine the prognostic effect of LMR on lung cancer. Our results demonstrated a significantly strong correlation between LMR and prognosis of lung cancer patients, and high LMR indicated longer PFS and OS compared with low LMR. Moreover, the same outcomes were found in different subgroups regardless of treatment, LMR cut-off value, and districts.

Subgroup analysis was performed to detect the potential heterogeneity among included studies (Table 2). The results showed that high LMR revealed a favorable PFS and OS in NSCLC patients. Additionally, no significant difference between LMR and OS was found in SCLC patients in this study, and similar results were reported by Hu et al. and Cao et al. [17, 22]. By contrast, Go et al. showed a different result, where high LMR also indicated better OS in SCLC [16]. However, the difference in survival was observed only in patients with limited-stage disease (ED), possibly leading to the difference in the results between that study and ours.

The subgroup analysis showed that high LMR indicated a better PFS and OS in patients who underwent surgery or not. However, no significant difference between LMR and OS in patients who did not receive operation was reported in Cao et al.'s study, which included the second largest number of cases (707) [17]. This result indicated that the potential roles of LMR must be verified in further research for this subgroup of patients.

Considering that the published studies used various cut-off values ranging from 2.62 to 4.56, our subgroup analysis indicated that the prognostic value of LMR was significant regardless of cut-off value. Moreover, the analysis of geographic area subgroup indicated that the prognostic value of LMR was observed in China and Korea. However, only one study involving 188 Korean patients was included in the meta-analysis; therefore, the conclusion of the study performed in Korea should be considered with caution, and more studies should be performed to verify the results.

Although the exact mechanisms have not yet been elucidated, either absolute lymphocyte or monocyte count is significantly associated with the prognosis of various cancers; moreover, LMR has been extensively reported and viewed as a promising prognostic indicator in malignancies [27–29]. Tumor-infiltrating lymphocytes are associated with a favorable prognosis in various tumors. By contrast, low lymphocyte count, which leads to inferior survival in multiple cancers, possibly results in an insufficient immunological reaction [30]. Moreover, tumor-associated macrophages (TAMs) derived from circulating monocytes recruit and constitute the inflammatory infiltrate and promote cancer proliferation, angiogenesis, and metastasis [31]. Studies have demonstrated that the infiltration of TAMs is associated with cancer survival, and the peripheral blood monocytes may reflect the formation or presence of TAMs [32, 33]. In reference to these factors, high LMR may reflect the inflammatory tumor microenvironment and inhibits tumor progression.

There are several limitations in this meta-analysis due to practical constraints. Firstly, a limited number of eligible studies exist. All articles included in this meta-analysis were conducted in Asian countries, and all publications used in data synthesis were published in English, suggesting that some eligible studies may have been excluded due to language constraints. Second, heterogeneity was significant in various pooled results. Inconsistent issues within each study, such as therapy options, tumor stage, or gender, may have caused such heterogeneity. Moreover, due to lack of background information, we failed to perform subgroup analyses on parameters such as gender or tumor stage. Additionally, the cut-off values used to define LMR, as mentioned, were inconsistent and thus we could not identify which cut-off value was the most reliable. Nonetheless, with the use of a detailed protocol and a carefully pooled data and random-effect model, the impact of heterogeneity was constrained to the minimum, and the pooled results were guaranteed reliable.

To conclude, our results demonstrated that elevated LMR results in a favorable outcome in lung cancer patients. Given that the use of LMR as indicator is facile and low cost, LMR is a potential marker for patient prognosis.

## MATERIALS AND METHODS

### Search strategies

We searched the PubMed, Embase, Web of Science, and the Cochrane Library databases to identify relevant studies published up to May 2017. Our search terms included “lung neoplasm” or “lung carcinoma” or “lung cancer” or “lung tumor” or “cancer of lung” or “lymphocyte to monocyte ratio” or “lymphocyte monocyte ratio” or “lymphocyte-to-monocyte ratio” or“LMR”. All relevant studies were included based on their titles and abstracts. Moreover, their reference lists were reviewed to identify other relevant articles.

### Inclusion criteria

The inclusion criteria were as follows: (1) the study evaluated the association between LMR and prognosis; (2) the study includes adequate data for calculation of the hazard ratio (HR) and their corresponding 95% confidence intervals (CIs); (3) the cut off value for LMR was reported; and (4) the articles published in English.

### Exclusion criteria

The exclusion criteria were as followed: (1) studies published as reviews, letters, comments, or case reports; (2) studies without adequate data for the calculation of HR and CI; (3) overlapped or duplicated data; and (4) studies published in languages other than English.

### Data extraction and quality assessment

Two reviewers (WL and GM) searched the manuscripts independently and then they compared the data they gathered; any dispute was settled by the third investigator. The following information were extracted: first author's name, year of publication, country, ethnicity, number of cases, demographic characteristics (e.g., patient age and gender), cut-off value, treatment, and prognosis.

Multivariate outcomes were extracted when both multivariate and univariate analyses were performed in the included studies. The Newcastle–Ottawa Scale (NOS) was used to evaluate the qualities of all the included studies [34]. This evaluation tool covered the selection, comparability, and clinical outcomes. Studies with a score of ≥ 6 were defined as high-quality studies.

### Statistical analysis

Survival data including progression-free survival (PFS) and OS, were reported in terms of HRs and 95% CIs. The heterogeneity of all the studies was assessed by Cochran's *Q* and Higgins *I*^2^ tests [35]. Random-effect model was used when the heterogeneity was significant (*p* < 0.05/*I*^2^ > 50%); otherwise, fixed-effect model was used [36]. Sensitivity analysis was performed to estimate the stability of the result by excluding individual studies. Moreover, Begg's tests was used to assess the presence of a potential publication bias [37]. If *p* value is no more than 0.05, it is considered statistical significance. All data analyses were performed using Stata 12.0 software (StataCorp LP, TX, USA).
